# Retinal morphology across the menstrual cycle: insights from the UK Biobank

**DOI:** 10.1038/s44294-024-00042-y

**Published:** 2024-11-08

**Authors:** Ana Paula Ribeiro Reis, Estelle Ioannidou, Siegfried Karl Wagner, Robbert Struyven, Zihan Sun, Paul Foster, Anthony P. Khawaja, Axel Petzold, Sobha Sivaprasad, Nikolas Pontikos, Pearse A. Keane, Konstantinos Balaskas, Elena Greco, Stamatina Iliodromiti, Praveen J. Patel

**Affiliations:** 1 National Institute for Health Research Biomedical Research Centre at Moorfields Eye Hospital NHS Foundation Trust and UCL Institute of Ophthalmology, London, UK; 2 Centre for Medical Image Computing, University College London, London, UK; 3 The Royal London Hospital, Barts Health NHS Trust, London, UK; 4 Wolfson Institute of Population Health, Queen Mary University of London, London, UK

**Keywords:** Health care, Diagnosis

## Abstract

Oestradiol and progesterone levels are higher in menstruating women than men of the same age, and their receptors are present in their neurosensory retina and retinal pigment epithelium. However, the impact of this hormonal environment on retinal physiology in women remains unclear. Using self-reported menstrual cycle phases as a surrogate for fluctuating hormonal levels, we investigated associations with retinovascular indices on colour fundus photograph and retinal thickness in optical coherence tomography across regularly menstruating women in the UK Biobank. We found no differences in retinal thickness across the cycle; however, vessel density, arteriolar and venular, and fractal dimension were higher in the luteal phase than follicular. The calibre of the central retinal vessels did not differ. This study suggests that the menstrual cycle phase might be associated with retinal microvasculature density in non-invasive imaging. It raises awareness for this understudied area, providing insights into neuroscience fields and epidemiological studies.

## Introduction

Oestradiol and progesterone are sex steroid hormones (SSH) found in both sexes, with higher levels in women during their reproductive years. For approximately four decades of a woman’s life^1,2^, dynamic production of ovarian SSH provides feedback to the hypothalamic-pituitary complex^3^. It acts not only as a regulator of sexual differentiation and reproduction but also on various systems, including brain structure and memory^4–8^. Both oestradiol and progesterone cross the blood-brain barrier and blood-retinal barrier^9^ and have receptors expressed in multiple ocular structures^10–12^, from the cornea to the retina^13,14^. Oestrogen receptor alpha (ORα) was first identified in the neurosensory retina and retinal pigment epithelium (RPE) in post-mortem eyes of young women^12^, and the discovery of oestrogen receptor beta (ORβ) and progesterone mRNA followed^10,11^.

A growing body of literature addresses poorly understood sex differences in retinal morphology, most frequently with optical coherence tomography (OCT). OCT is a non-invasive technique that displays each retinal layer using backscattered infrared light wave reflectance, providing cross-sectional retinal scans^15^. In OCT studies, women consistently exhibit a broader and shallower foveal pit and thinner macular thickness in the central and inner 3 mm macular zone compared to men^16–18^. Women also have a higher prevalence of idiopathic macular holes and a potentially lower risk of primary open-angle glaucoma (POAG) and age-related macular degeneration (AMD) with prolonged oestradiol exposure^19–25^. Such disparities in retinal structure and disease prevalence between women and men highlight that still unknown variables must drive such differences. Understanding the impact of fluctuating SSH in women’s retinas could be crucial in comprehending these asymmetries.

Research on retinal morphology throughout the menstrual cycle is limited^26–33^, with most studies focusing on choroidal thickness, a vascular layer underneath the retina. They report consistently thinner choroidal thickness (CHT) during the luteal phase compared to the follicular phase. Still, the combined sample size from the CHT studies amounts to only 74 women^26,27,30^ reflecting the generally small sample sizes in this field. OCT studies examining macular layers show no significant differences between menstrual cycle phases, though the number of studies addressing this question is even more limited than for CHT28,30. In recent years, menstrual cycle tracking applications and wearables have expanded the available data on menstrual cycles, allowing large epidemiologic studies to be conducted with millions of data points^34–39^. However, a knowledge gap persists on how hormonal fluctuations, as tracked by these tools, interact within the physiology of women^34–39^.

We present the first cross-sectional analysis of eumenorrheic women in the UK Biobank (UKBB) cohort relating ocular variables to the menstrual cycle. We investigated macular structural differences on OCT and retinovascular indices on colour fundus photography (CFP) at the follicular and luteal phases of the menstrual cycle.

## Results

The UKBB is a foundational resource for epidemiological and ophthalmic research, showcasing diverse participant demographics, ocular health metrics, and systemic health indicators of a predominantly healthy subset of the UK population. To explore retinal morphology across the menstrual cycle, we identified the 44,534 female participants who underwent ocular assessments using OCT and CFP modalities. We isolated the subgroup of eumenorrheic women from this cohort based on multiple exclusion criteria outlined in the methods section. We then fitted multivariable linear mixed-effects models to investigate associations between menstrual cycle phases and retinal morphological features on retinal imaging. The results presented in the following sections provide insights into those associations.

### 2278 eumenorrheic Women in UKBB

**Fig. 1:**
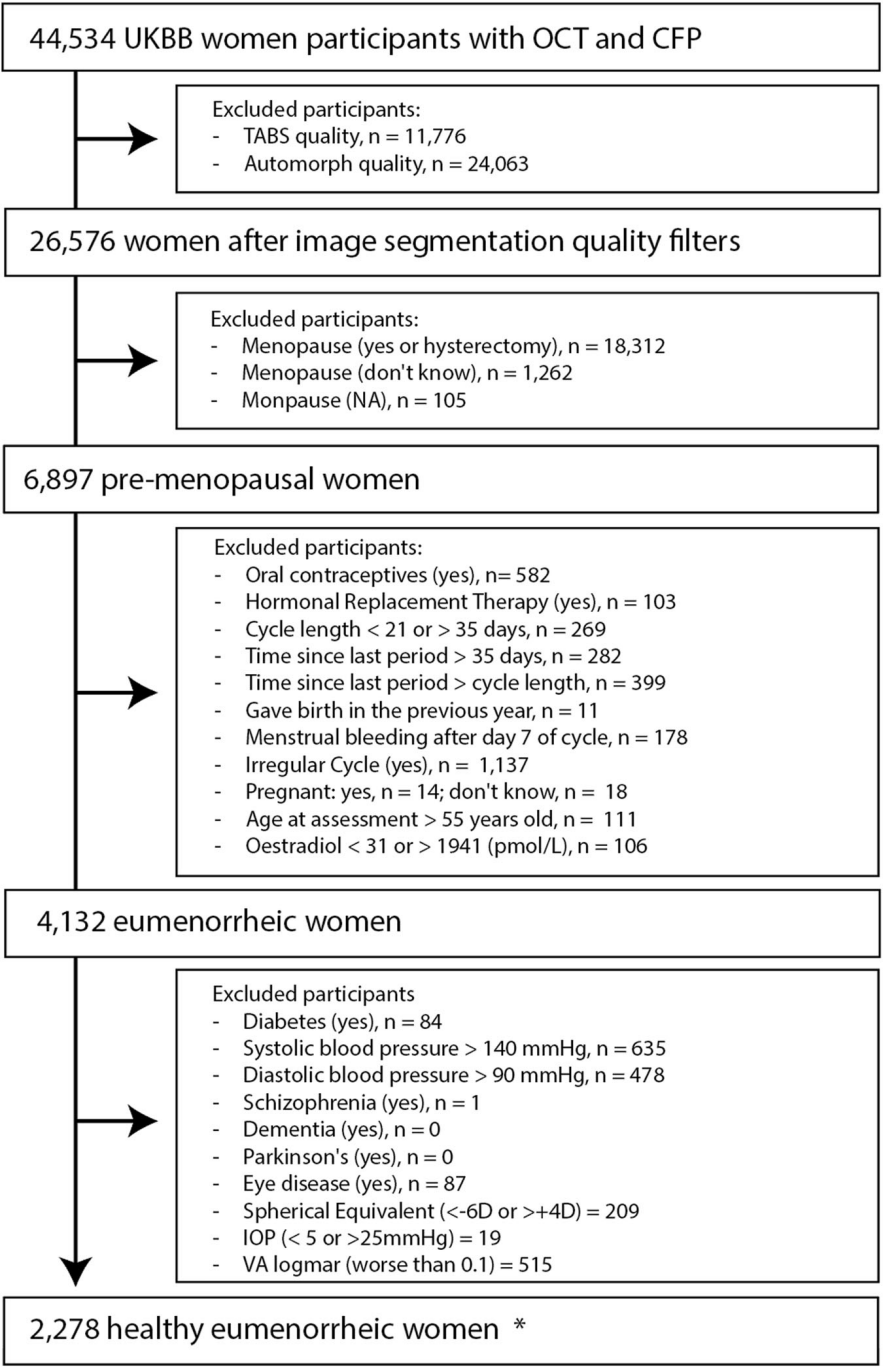
Flowchart detailing inclusion and exclusion of participants in UKBB. The starting point consisted of women included in either of the two ophthalmic assessments who underwent retinal imaging (OCT and CFP). When participants attended both ophthalmic assessments, the earliest one was preferred. The same participant might be excluded in different categories within each exclusion box. * “Healthy eumenorrheic women”, refers to the final subset of women, excluding participants with specified health conditions and criteria that could interfere with the analysis.

In total, 44,534 women underwent either of the two UKBB ophthalmic assessments and had retinal imaging (OCT and CFP) taken. We initiated the selection process by excluding scans with poor image quality at the eye level. The exclusion of an OCT scan resulted in excluding the same eye CFP, and vice versa, to allow a full-case analysis. 26,576 women had at least one eye with OCT and CFP of sufficient quality for analysis. After the removal of unknown menopausal status and post-menopausal women, we identified 6897 pre-menopausal participants. Furthermore, we removed women not experiencing a spontaneous natural eumenorrheic cycle, based on questionnaire information and oestradiol levels^40^, counting 4132 eumenorrheic women. Ultimately, participants exhibiting ocular and systemic features potentially influencing retinal morphology were excluded, and 2278 eumenorrheic women could be included for analysis. Figure 1 details the inclusion/exclusion criteria in the form of a flowchart. All UKBB fields used for the analysis are detailed in Supplementary Table 1.

### Oestradiol curve supports questionnaire-estimated menstrual phase

Among our cohort of 2278 eumenorrheic women, a subset of 1677 women had serum oestradiol levels measured on the same day of questionnaire and retinal imaging. We used this subset to validate our questionnaire-based follicular and luteal phase estimation. Based on self-report estimation, 1167 women with serum oestradiol levels were in the follicular phase, and 510 were in the luteal phase. Table 1 presents the average cycle day and oestradiol levels for both groups. Although oestradiol reference values exhibit considerable individual variability^41^, we observed a significant difference in the oestradiol profiles between our follicular and luteal groups (*p* < 0.01), with average levels (±SD) of 569.4 (±354.9) pmol/L and 510.7 (±302.5) pmol/L, respectively. Both groups were within the normal oestradiol reference range for menstruating women in each phase^41^.

**Table 1:** Day of the cycle as answered in the UKBB self-report field “time since last menstruation” in days and serum oestradiol levels in pmol/L in the estimated follicular and luteal phase groups

Characteristics		Cycle phase		
		Follicular (*n* = 1167)	Luteal (*n* = 510)	*p* value
Time since last menstruation, mean ± SD (range)	Days	9 ± 5 (1, 21)	21 ± 3 (15, 35)	<0.01 *
Serum oestradiol, mean ± SD (range)	pmol/L	569.4 ± 354.9 (175.1, 1938.7)	510.7 ± 302.5 (176.8, 1906.0)	<0.01 *

Statistical significant differences between the groups (*p* < 0.05) are highlighted in bold. Both variables significantly differed between groups and are within the normal range for menstruating women.

*n* sample size, *SD* standard deviation.

**Fig. 2:**
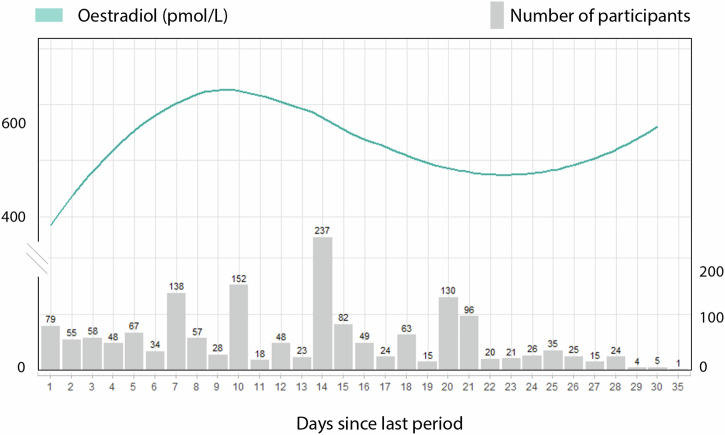
Mean oestradiol levels per day of the cycle in our UKBB sample. Oestradiol levels in pmol/L are represented on the curved line of the left y-axis. The number of participants who had hormone readings for each cycle day is shown on the right y-axis. Due to the small sample size, information is limited for individuals on cycle days beyond 29.

We plotted the average value for oestradiol per day of the cycle in Fig. 2 to understand its hormonal fluctuation within our cohort. The grey bars represent the available data points for each day of the cycle. Oestradiol reference levels published elsewhere show an increase in Oestradiol from the early follicular (31–771 pmol/L) to the late follicular phases (104–1742 pmol/L), reaching their highest at ovulation (275–2864 pmol/L). Following their ovulatory peak, they begin to decrease in the early luteal phase (95–1188 pmol/L) but remain elevated during the mid-luteal (151–1941 pmol/L) before gradually declining again in the late luteal phase (39–1769 pmol/L)^40^. Despite our cross-sectional study design, we observed similarities to the physiologic oestradiol curves^40^. In our cohort, oestradiol levels increase in women in the early days of the cycle, achieving their peak on day 10, to decline and achieve another plateau on the luteal phase. Fewer participants reported being on days 29–35 of the cycle, impacting those average values. Note that our methods are powered to identify women in the follicular and luteal phases, excluding the ovulatory phase.

### Demographics of women in the follicular and luteal phases

The 2278 eumenorrheic women analysed had a mean age of 45 ± 3 years and predominantly identified as White (88.7%), followed by individuals of other ethnicities (4.8%), Black (3.5%), and South Asian (3.0%). For a small percentage (0.04%), ethnicity data was unavailable. The average cycle length was 27 ± 3 days, with most participants reporting a cycle between 25 and 30 days (*n* = 1824). Participants were divided by cycle phase, with 1584 individuals (2568 eyes) estimated to be in the follicular phase and 694 participants (1124 eyes) in the luteal phase. The demographic characteristics of each group are presented in Table 2. All characteristics were assessed at the participant level. The groups were well-balanced, demonstrating no statistically significant differences in composition, except for time since the last menstruation, whose values were related to their corresponding cycle phase. Follicular participants reported a “time since last menstruation” of 9 (±5) days, and luteal phase participants 22 ( ± 3) days. Socio-economic status divided into quintiles was borderline significant between follicular and luteal participants (*p* = 0.06), with Follicular phase participants being negligibly less deprived (higher Index of Multiple Deprivation).

**Table 2:** Baseline characteristics according to cycle phase

Characteristics (participant level)		Cycle phase		
		Follicular (*n* = 1584)	Luteal (*n* = 694)	*p* value
Age, mean ± SD (range)	Years	45 ± 3 (40, 55)	45 ± 4 (55, 40)	0.18
Time since menarche, mean ± SD (range)	Years	32.0 ± 3.7(21, 45)	32.3 ± 3.8 (24, 43)	0.08
Time since last menstruation, mean ± SD (range)	Days	9 ± 5 (1, 21)	22 ± 3 (15, 35)	<0.01 *
Ethnicity, n (%)	Black	46 (2.90)	33 (4.76)	0.24
	Other	75 (4.73)	34 (4.90)	
	South Asian	50 (3.16)	18 (2.59)	
	White	1,412 (89.14)	609 (87.75)	
Socioeconomic status, mean ± SD	1st quintile	5.06 ± 1.40	4.97 ± 1.40	0.06
	2nd quintile	9.45 ± 1.22	9.26 ± 1.19	
	3rd quintile	14.34 ± 1.64	14.51 ± 1.80	
	4th quintile	22.19 ± 2.63	22.18 ± 2.72	
	5th quintile	38.21 ± 8.85	37.20 ± 7.77	
BMI, mean ± SD (range)	kg/m^2^	25.30 ± 4.38 (16.58, 43.33)	25.35 ± 4.34 (17.36, 46.66)	0.57
Smoking, mean ± SD (range)	Pack years	13.38 ± 10.16 (0.00, 64.00)	13.17 ± 9.36 (0.15, 48.00)	0.83

*n* sample size, *SD* standard deviation, *BMI* body mass index (kg/m^2^). Statistical significant differences between the groups (*p* < 0.05) are highlighted in bold.

### Univariate analysis shows no significant associations between macular variables and the menstrual cycle phase

**Fig. 3:**
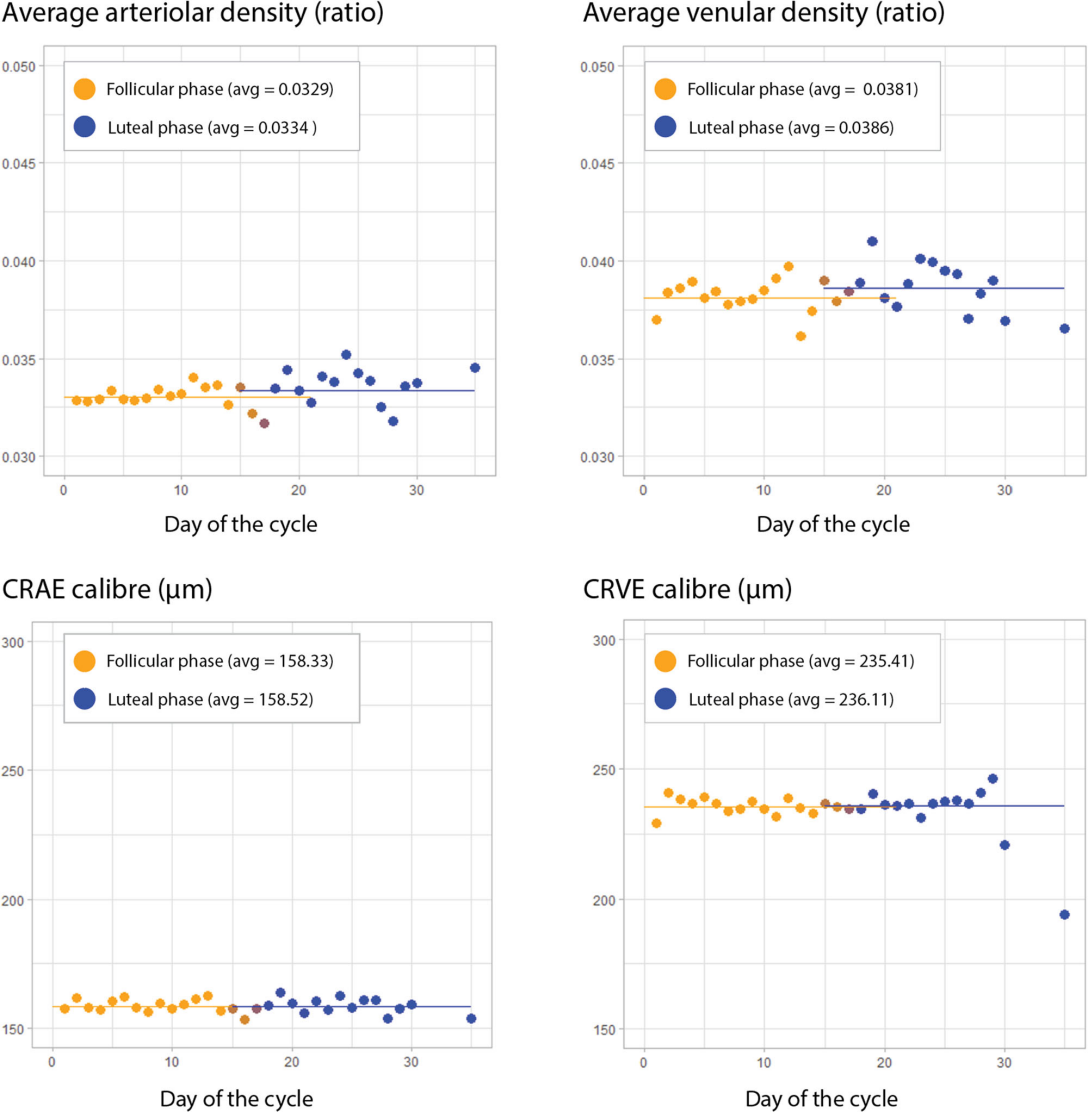
Distribution of arteriolar and venular perfusion densities and CRAE and CRVE calibres across different days of the menstrual cycle. Participants were grouped based on the reported cycle day, and ocular measurements were initially averaged for each participant’s eyes per day. The top graphs show density (microvasculature), and the bottom graphs show central vessel calibre (macrovasculature). The left-side graphs portray arteriolar parameters, while the right-side graphs present venular measurements. The scale was maintained within both density and calibre graphs to allow comparisons of arterial and venous parameters. Follicular phase measurements are dotted in orange, and luteal phase measurements are dark blue. CRAE Central Retinal Artery Equivalent, CRVE Central Retinal Vein Equivalent, avg average

For the univariate analysis of CFP and OCT macular variables, both eyes of each participant were initially averaged at the individual level. There were no significant associations (*p* > 0.05) between macular variables and the follicular or luteal phases. These results are presented in Table 3. The distribution of average arteriolar and venular perfusion density, central retinal artery equivalent (CRAE) and central retinal vein equivalent (CRVE) per day of the cycle are portrayed in Fig. 3. Both density and calibre of venular parameters were higher than arteriolar (*p* < 0.01).

**Table 3:** Univariate analysis of retinovascular indices and total retinal thickness according to cycle phase

Characteristics (participant level)		Cycle phase		
		Follicular (*n* = 1584)	Luteal (*n* = 694)	*p* value
Retinovascular Indices, mean (±SD)	CRAE, μm	158.33 ± 17.49	158.52 ± 17.67	0.77
	CRVE, μm	235.41 ± 22.99	236.11 ± 22.17	0.39
	Arteriolar density, ratio	0.03 ± 0.01	0.03 ± 0.01	0.06
	Venular density, ratio	0.04 ± 0.01	0.04 ± 0.01	0.14
	Fractal dimension	1.50 ± 0.03	1.50 ± 0.03	0.06
Retinal Thicknesses, mean (±SD) ^*^	TRT, μm	327.44 ± 16.47	328.80 ± 21.21	0.43
	IRT, μm	138.92 ± 13.69	139.45 ± 14.93	0.50
	ORT, μm	188.31 ± 15.22	189.12 ± 18.71	0.78

Results are shown at the individual level. Retinal layer thicknesses and retinovascular indices from both individual’s eyes were averaged.

* Retinal thickness metrics are detailed as the averages within the 3-mm Early Treatment Diabetic Retinopathy (ETDRS) circle.

*n* sample size, *CRAE* Central Retinal Artery Equivalent, *CRVE* Central Retinal Vein Equivalent, *SD* standard deviation.

### Increased vascular perfusion and fractal dimension on the luteal phase in multivariate analysis

The multivariate analysis was adjusted to age, ethnicity, deprivation, body mass index (BMI), and smoking. Table 4 shows all coefficients and their significance. The linear mixed effects model results are presented as differences per standard deviation (per SD) to facilitate comparing results between units due to their small absolute numbers. The luteal phase was associated with an increased perfusion density in both arterioles (0.20, 95% CI: 0.03, 0.38, *p* = 0.03) and venules (0.21, 95% CI: 0.03, 0.38, *p* = 0.02). Fractal Dimension was also increased in the luteal phase (0.19, 95% CI: 0.01, 0.38, *p* = 0.05). CRAE and CRVE were not significantly different between phases. Age was associated with a slight reduction in all retinovascular indices except CRVE. On the other hand, Black ethnicity was associated with an increase in CRAE and CRVE.

**Table 4:** Association between retinovascular indices derived from colour fundus photographs with cycle phase, adjusted for covariates and estimated through multivariable linear mixed-effects models

Characteristics		CRAE, μm		CRVE, μm		Arteriolar density, ratio		Venular density, ratio		Fractal dimension	
		Difference per SD (95% CI)	*p* value	Difference per SD (95% CI)	*p* value	Difference per SD (95% CI)	*p* value	Difference per SD (95% CI)	*p* value	Difference per SD (95% CI)	*p* value
Cycle Phase	Follicular	Reference		Reference		Reference		Reference		Reference	
	Luteal	0.12 (–0.04, 0.30)	0.16	0.13 (–0.04, 0.30)	0.14	0.20 (0.03, 0.38)	0.03*	0.21 (0.03, 0.38)	0.02*	0.19 (0.01, 0.38)	0.05*
Age	Years	–0.03 (–0.03, 0.02)	0.01*	–0.01 (–0.03, 0.02)	0.66	–0.04 (–0.06, –0.02)	<0.01*	–0.02 (–0.05, –0.00)	0.04*	–0.03 (–0.06, –0.01)	0.01*
Ethnicity	Black	0.75 (0.32, 1.35)	<0.01*	0.84 (0.32, 1.35)	<0.01*	0.06 (–0.48, 0.60)	0.83	0.41 (–0.13, 0.94)	0.14	0.03 (–0.54, 0.59)	0.93
	Other	0.04 (–0.16, 0.45)	0.79	0.15 (–0.16, 0.45)	0.35	0.03 (–0.30, 0.35)	0.88	0.19 (–0.13, 0.51)	0.24	0.02 (–0.31, 0.36)	0.89
	South Asian	–0.20 (–0.48, 1.43)	0.68	0.47 (–0.48, 1.43)	0.33	–0.64 (–1.48, 0.20)	0.14	–0.36 (–1.21, 0.50)	0.42	–0.82 (–1.69, 0.06)	0.07
	White	Reference		Reference		Reference		Reference		Reference	
IMD	deciles	0.01 (–0.01, 0.01)	0.02*	0.00 (–0.01, 0.01)	0.84	0.00 (–0.00, 0.01)	0.30	0.00 (–0.01, 0.01)	0.88	0.00 (–0.01, 0.01)	0.97
BMI	kg/m^2^	–0.01 (–0.01, 0.03)	0.16	0.01 (–0.01, 0.03)	0.44	–0.02 (–0.04, 0.00)	0.10	0.00 (–0.02, 0.02)	0.72	–0.01 (–0.03, 0.01)	0.51
Smoking	Pack Years	0.01 (0.00, 0.02)	0.20	0.01 (0.00, 0.02)	0.01*	0.00 (–0.01, 0.01)	0.61	0.00 (–0.01, 0.01)	0.46	0.00 (–0.01, 0.01)	0.94

* Statistically significant associations at significance level = 0.05. Statistical significant differences between the groups (*p* < 0.05) are highlighted in bold.

*CRAE* Central Retinal Artery Equivalent, *CRVE* Central Retinal Vein Equivalent, *SD* standard deviation, *CI* confidence interval, *IMD* Index of Multiple Deprivation, *BMI* body mass index (kg/m^2^).

### Retinal thickness was not associated with the cycle phase

Adjusted for confounders, the cycle phase did not show any association with TRT, IRT or ORT, averaging all measurements within the 5 Early Treatment Diabetic Retinopathy *(*ETDRS) 3 mm ring inner-sectors. There were no relevant associations from the covariates. Table 5 displays this information.

**Table 5:** Association between OCT-derived total retinal, inner retinal, and outer retinal thickness with cycle phase, adjusted for covariates and estimated through multivariable linear mixed-effects models

Characteristics		TRT		IRT		ORT	
		Thickness difference, μm (95% CI)	*p* value	Thickness difference, μm (95% CI)	*p* value	Thickness difference, μm (95% CI)	*p* value
Cycle phase	Follicular	Reference		Reference		Reference	
	Luteal	1.57 (–1.82, 4.94)	0.37	1.12 (–2.13, 4.35)	0.51	1.19 (–2.22, 4.60)	0.50
Age, mean	Years	–0.02 (–0.46, 0.43)	0.94	0.12 (–0.30, 0.54)	0.58	0.03 (–0.42, 0.48)	0.89
Ethnicity	Black	–8.99 (–19.2, 1.23)	0.09	–4.47 (–14.33, 5.39)	0.38	–3.52 (–13.93, 6.89)	0.51
	Other	–2.02 (–8.09, 4.03)	0.52	1.53 (–4.29, 7.35)	0.61	–2.51 (–8.64, 3.62)	0.43
	South Asian	–0.89 (–17.02, 15.24)	0.91	–2.07 (–16.99, 12.85)	0.79	2.50 (–12.93, 17.94)	0.75
	White	Reference		Reference		Reference	
IMD	deciles	–0.01 (–0.13, 0.12)	0.91	0.03 (–0.09, 0.15)	0.68	0.01 (–0.12, 17.94)	0.93
BMI	kg/m^2^	–0.20 (–0.57, 0.18)	0.30	0.04 (–0.32, 0.40)	0.82	–0.40 (–0.78, –0.01)	0.04*
Smoking	Pack Years	–0.06 (–0.23, 0.11)	0.48	–0.15 (–0.31, 0.01)	0.07	0.10 (–0.07, 0.27)	0.24

* Statistically significant associations at significance level = 0.05. Statistical significant differences between the groups (*p* < 0.05) are highlighted in bold.

*SD* standard deviation, *CI* confidence interval, *IMD* Index of Multiple Deprivation, *BMI* body mass index (kg/m^2^), *TRT* total retinal thickness, *IRT* inner retina thickness, *ORT* outer retina thickness.

Considering the five central subfields of the 3 mm inner ETDRS ring individually, there were no associations with TRT, IRT, or ORT. The central subfield was not calculated for IRT due to the absence of this layer. Likewise, it was not included in the TRT, as it encompasses the IRT. These results can be found in the Supplementary Table 2.

## Discussion

This is the largest report on OCT and the first CFP study assessing associations between menstrual cycle phases and retinal morphology to date. OCT and CFP are non-invasive imaging modalities that access the central nervous system and microvasculature, capable of detecting systemic nuances in these structures^42^. We assembled a cohort of women from the UKBB, a large prospective study representative of the UK population, where we controlled our analysis for ethnicity and general health parameters such as BMI, smoking, and deprivation. Additionally, we used various methods to detect and exclude significant sight-involving conditions and other prevalent diseases known to impact the retina. We found higher retinal arteriolar density, venular density, and fractal dimension on CFP during the luteal phase than in the follicular phase, with preserved central retinal artery and vein calibres.

The UKBB offers a unique opportunity to investigate ocular morphology within a large cohort of adults aged 40 years and older. Despite being in their last reproductive decade, 2278 female participants reported being on their menstrual cycle and eligible for our analysis. We remotely accessed UKBB retinal imaging files and utilised automated segmentation techniques to derive retinovascular indices and macular thickness measurements from CFP and OCT, exploring their associations with the menstrual cycle and questionnaire information within UKBB.

The CFP parameters were calculated using a deep learning algorithm, AutoMorph. It binarises the CFP to calculate vessel density and distinguishes vessel pixels from background pixels^43^. An example of an AutoMorph mask can be found in Fig. 5b in the methods section. The higher luteal vessel perfusion density indicates a greater number of pixels attributed to vessels in that phase, whether due to enhanced retinal vessel visibility, higher contrast, small-vessel calibre disparities or differences in fundus tessellation (variation in the visibility of large choroidal vessels). The increase in the luteal fractal dimension suggests an increase in the measurable branching of vessels in CFP and supports the previously mentioned associations. CRAE and CRVE were measured using the Knudtson formula within AutoMorph, and their lack of association with the cycle phase suggests that the menstrual hormonal fluctuations might not affect the macro retinal vessels.

One of the most consistently observed effects of oestrogen and progesterone is differences in vessel tone, with data on the effect of progesterone being more limited than oestrogens^44^. Oestradiol, the most potent oestrogen in the human body, is a potent vasodilator, enhancing beta-adrenergic receptor-mediated vasodilation, counteracting alpha-adrenergic vasoconstriction^45^ and impacting nitric oxide production and activity^46^. We report an increase in vessel density in the luteal phase, a period characterised by higher progesterone levels; however, we cannot comment on their levels within our cohort since progesterone serum levels are not part of the UKBB protocol. Progesterone has been shown to enhance both transcriptional and non-transcriptional nitric oxide synthesis at the cellular level, which may impact the microvasculature but not macrovasculature^47,48^. Studies using mice models have suggested that progesterone directly upregulates endothelial nitric oxide synthase expression in a dose-dependent and time-dependent manner^49^ and might have synergistic vasodilatory effects when added to oestrogens^50^. Angiogenesis, the process of new microvessel formation from a pre-existing vascular bed stimulated by various growth factors, also plays a vital role during the menstrual cycle in the endometrium^51^. Whether this might occur elsewhere in the body on a smaller scale is unknown.

Another way to study vessel perfusion density is through OCT angiography (OCTA), which was not part of the UKBB protocol. OCTA utilises differences in backscattered OCT signals to differentiate blood flow locations from static tissue locations and construct a retina angiogram^52^. Guo et al. enroled 62 women aged 27 (±2 SD) years to investigate OCTA in early follicular, ovulatory and luteal phases. They controlled for ovulation through urinary luteinising hormone monitoring at home and adjusting the analysis to age, mean arterial pressure, spherical equivalent, axial length, and intraocular pressure (IOP). They found no significant difference in vessel density of the superior capillary plexus. However, they did observe that vascular density in the deep capillary plexus within the nasal and inferior subfields was significantly lower during ovulation compared to the follicular or luteal phases^29^, suggesting fluctuations in microvasculature along the cycle. Another study found no significant differences in vessel density of the superficial or deep capillary plexus between the follicular, ovulatory and mid-luteal phases without performing any adjustments^33^. Additional studies exploring hemodynamic differences across the menstrual cycle with retrobulbar Doppler have yielded conflicting results. Karadeniz et al. conducted a study on 23 healthy women, examining retrobulbar circulation with serial colour Doppler ultrasonography during a normal menstrual cycle, and found that hemodynamic parameters remained stable throughout the menstrual cycle^53^. Another study by Haneda et al. describes a decrease in choroidal blood flow velocity during the late follicular phase, indicating a potential vessel choroidal calibre increase in this phase^54^.

Both our univariable and adjusted multivariable analyses did not reveal any differences in TRT, IRT, or ORT. Similar results were obtained in other studies with longitudinal designs. Ulaş et al. monitored 23 healthy nulliparous females with a mean age of 26 (±3 SD) years. They conducted OCT scans during the follicular, ovulatory, and mid-luteal phases within one menstrual cycle and found no statistical differences in TRT or RNFL^30^. Fortepiani et al. tracked 28 menstruating women with a mean age of 26 (±5 SD) years. They observed 16 of these women during the first and second halves of the menstrual cycle and the remaining 12 women over two or three cycles. Similarly, they found no statistical differences in foveal thickness between the follicular and luteal phases on OCT^28^. However, such associations can only be compared considering the study’s design and limitations. Retinal thickness is known to vary within the healthy population and between men and women18. While the large sample size from the UKBB helps to balance these natural variations, as this was a cross-sectional study, it may not capture individual phase-specific fluctuations, and these results cannot be extrapolated to a longitudinal design.

There are several other limitations to our study. Firstly, UKBB participants UKBB had only two ophthalmic visits to date. Due to the age of the cohort (over 40 years old), the number of menstruating women was small, and it was not possible to assess them longitudinally. Because of the age of the women included, we could only include women in their last reproductive decade, where menstrual cycle fluctuations become more irregular due to approaching menopause^36^. Secondly, the UKBB lacks detailed questionnaire information on lactation or hormonal contraception beyond oral methods, not including data on hormonal subcutaneous implants, injections, or hormonal intrauterine systems. Consequently, we could not identify and exclude women using these methods. Thirdly, since crucial hormones such as luteinising hormone or progesterone were not part of the UKBB protocol and not all women had serum estradiol measurements, it was not feasible to combine self-reported cycle phase estimation with hormonal assessments for this cohort. Self-reported menstrual data may suffer from recall bias, inconsistent tracking, lack of standardisation, and irregularity, all of which could limit the accuracy of cycle phase estimation and the interpretation of hormonal influences. Integrating cycle monitoring, self-reporting, and hormonal levels collected multiple times over the cycle in a longitudinal approach at similar times of the day would provide a stronger methodology^41,55,56^. Although we divided participants based on answers that did not suggest being in the ovulatory phase, it could not be reliably excluded based solely on self-reports, and we were also unable to differentiate women in subphases such as early and late follicular.

We encourage further research in this field, particularly studies with a longitudinal design that involve repeated retinal imaging of the same cohort across different menstrual cycle phases. Participants should be instructed on cycle tracking a couple of months before the beginning of the study to familiarise them with the method since it’s not encouraged to assume that the cycle is 28 days41,56,57. Multiple methods exist for reporting menstrual cycle phases, and it is recommended to combine objective evaluations, such as using peak luteinising hormone levels to predict ovulation, with self-reported data 41,56,57. To better understand the relationships of SSH and retinal morphology, serum measurements of oestradiol and progesterone on the day of retinal imaging would also be beneficial.

Finally, despite all these challenges, studies on the interaction between female SSH and the retina should not be dismissed. Our study suggests that menstrual cycle associations might be found on the level of retinal microvasculature, which could inform neuroscience studies and epidemiological insights into retinal physiology and pathophysiology. For decades, it was argued that women’s hormonal fluctuations confounded scientific work. This led to the exclusion of women from research^8,57^ and affected the generalizability of findings^58,59^. Further studies are required to elucidate the effects of the menstrual cycle on the retina, especially in a longitudinal fashion.

In conclusion, we observed higher physiological retinal vessel density during the luteal phase, indicating microvasculature alterations compared to the follicular phase. Despite facing limitations such as cross-sectional design, age-related cohort factors, and challenges in defining cycle phases, our findings emphasise the need for further research in this domain. Addressing these challenges could help tailor retinal normative ranges for women, improve understanding of normal physiology and shed light on epidemiological differences in sex-specific retinal characteristics.

## Methods

### Design, participants, and setting

The UKBB is a prospective, population-based, multi-centric cohort data resource with approximately 500,000 participants aged 40–69 years at recruitment, residing in the United Kingdom and registered with the National Health Service (NHS). The North West Multi-centre Research Ethics Committee approved the study (reference no., 06/MRE08/65), in accordance with the principles of the Declaration of Helsinki. Detailed information about the study is available on the UKBB website (www.ukbiobank.ac.uk). The study protocol incorporated various research modalities, including questionnaires, verbal interviews, physical measurements, and blood assessments conducted over multiple visits. Additionally, a subset of participants underwent comprehensive ocular assessments with retinal imaging at two visits in six acquisition centres (Sheffield, Liverpool, Birmingham, Croydon, Hounslow, and Swansea).

The reporting adheres to the guidelines established by the Strengthening the Reporting of Observational Studies in Epidemiology^60^ and aligns with the recommendations outlined in the Advised Protocol for OCT Study Terminology and Elements^61^.

### Ophthalmic assessment

The first ocular visit (Instance 0) in the UKBB occurred from June 2009 to July 2010 and included 67,681 participants. The first repeat ocular visit (Instance 1) occurred between August 2012 and June 2013 and included 19,346 participants, including newly recruited and follow-up individuals. Visual acuity (VA) was assessed using a logarithm of the minimum angle of resolution (LogMAR) chart (Precision Vision, LaSalle, Illinois, USA) displayed on a computer screen under standard illumination with distance correction. Non-cycloplegic autorefraction was conducted using the Tomey RC-5000 Auto Refkeratometer (Tomey, Nagoya, Japan), and the spherical equivalent was computed by adding the sum of the spherical power and half of the cylindrical power. Corneal compensated IOP was determined using the Ocular Response Analyzer (ORA) (Reichert, Philadelphia, Pennsylvania, USA)^62^. Retinal imaging for this analysis encompassed two modalities: OCT and non-mydriatic CFP^62,63^. The OCT 3D raster scan protocol included a 6x6mm area centred on the fovea, consisting of 128 B-scans and an axial resolution of 6 μm (OCT-1000 Mark II, Topcon, Japan). CFP was captured with a digital camera on the OCT-1000 Mark II (Topcon, Japan) with a 45° field angle, centred on the fovea and included the optic disc.

### Inclusion and exclusion criteria

All eyes of women who had retinal imaging performed as part of the UKBB study and had not withdrawn consent as of September 5, 2023, were used as a starting point for this analysis. We included only one visit per participant and included both eyes when available. Patients recruited at either Instance 0 or Instance 1 were included. If participants attended both instances, only the first was considered, and if there were multiple measures on the same day, the one with the highest quality was chosen. Eyes with OCT segmentation quality in the bottom 20th percentile, as indicated by the TABS indicator, and those with poor CFP quality based on AutoMorph’s quality metric were excluded. The exclusion of an OCT scan resulted in excluding the same eye CFP, or vice versa, to allow a full-case analysis. The exclusion of both study eyes led to the exclusion of the participant.

Menstrual and reproductive history was gathered via self-administered questionnaires. The analysis excluded women who reported to be in post-menopause, were unsure of their menopausal status or had a hysterectomy. Additionally, pre-menopausal women using oral contraceptives or hormonal replacement therapy, pregnant, within one year of childbirth or with irregular menstrual cycles were not considered. Participants with cycles shorter than 21 days or longer than 36 days were excluded. Furthermore, those whose last period had occurred more than 36 days ago or who were experiencing menstrual bleeding after day 7 of the cycle at the time of the study were also disregarded. Using the available serum oestradiol data, we excluded the participants with oestradiol levels lower than 31pmol/L, indicating menopause, and exceeding 1941 pmol/L, values that are in the exclusive range of ovulation or pregnancy. Our study compares women in the follicular and luteal phases, avoiding estimating the ovulatory phase due to its unreliability when based on self-report^41^.

Finally, systemic disease impacting posterior pole morphology, based on validated definitions of prevalent disease in the UKBB, led to the exclusion of individuals with diabetes mellitus and neurodegenerative disease^64–67^. Participants with average systolic blood pressure ≥140 mmHg or diastolic ≥90 mmHg were excluded^68^. Finally, those self-reporting glaucoma, diabetes-related eye disease, eye injury, cataract, macular disease, or other severe eye conditions were not included^18^. High refractive error (> +4 dioptres [D] or < -6D), poor visual acuity (VA) (logmar worse than 0.1 [20/25 Snellen equivalent]), and Goldmann-corrected intraocular pressure (IOP) ≥ 25 mmHg or ≤ 5 mmHg were not considered^18^.

### Exposure variables

**Fig. 4:**
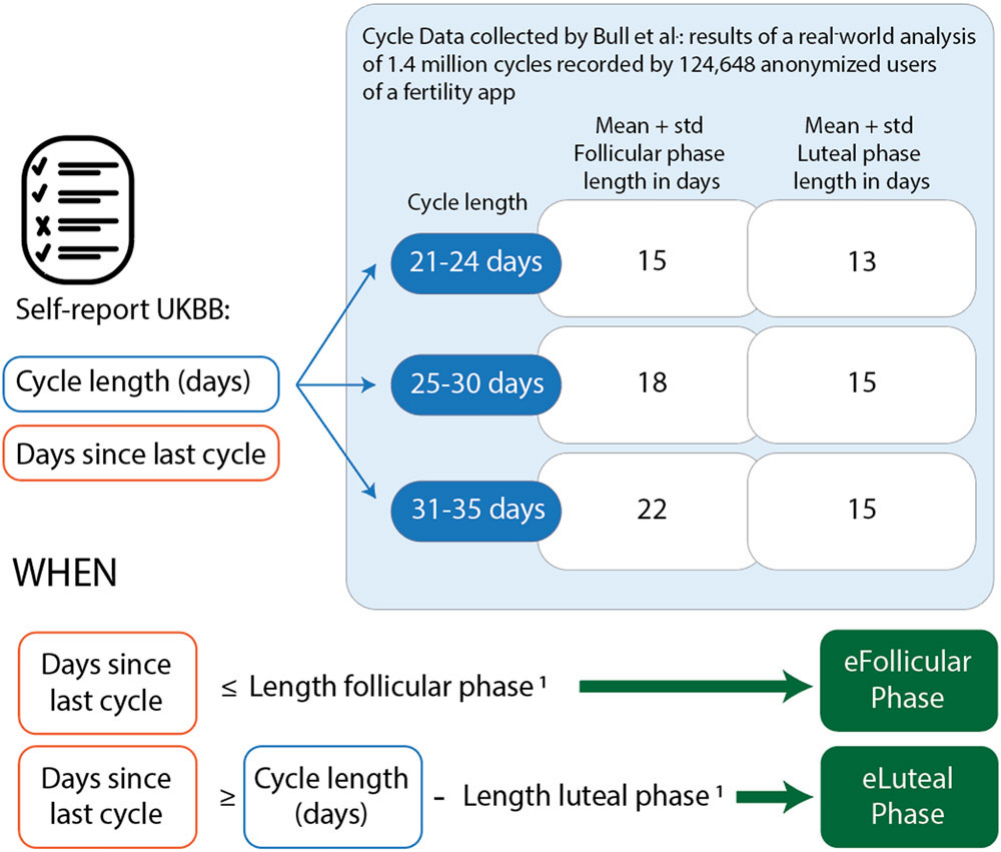
Framework for estimating menstrual cycle phases based on self-reported questionnaire information from the UKBB. Participants were categorised into three groups based on reported cycle length: 21–24 days, 25–30 days, and 31–35 days. Follicular and luteal phases were estimated based on the day of the cycle reported by the participants and the duration of the phases reported by Bull et al.^36^ in a large epidemiological study based on app-reported cycle data^1^. The length of each phase reported by Bull et al.^36^ was used to set thresholds according to the reported cycle length, which were applied to “days since the last cycle.”

Cycle length in days, and days since the last period were self-reported at the time of the ocular exam and serum oestradiol measurement. The menstrual cycle phase was determined by aligning questionnaire answers with the results of a real-world analysis of 1.4 million cycles recorded by 124,648 anonymised users of a fertility app^36^. UKBB participants were categorised into three groups based on the duration of their menstrual cycles: 21–24, 25–30, and 31–35 days, as reported by Bull et al.^36^. Within each cycle length category, individuals were further classified into follicular or luteal phases. For the follicular phase, the lower boundary was set at one day. The upper boundary was determined as the mean plus one standard deviation of the follicular phase length, as reported by Bull et al.^36^. For the luteal phase, the lower boundary was calculated as the participant’s cycle length minus the mean plus one standard deviation of the luteal phase length reported in the same study^36^. In contrast, the upper boundary was the participant’s cycle length. A graphic representation of this framework is depicted in Fig. 4.

### Outcome variables

OCT B-scans underwent segmentation using the *Topcon Advanced Boundary Segmentation* (TABS) software^69^. This software utilises dual-scale gradient information for retina layer segmentation and has been previously validated on the UK Biobank^18,70^. TABS provides thickness values between nine retinal boundaries on a B-scan level and *en-face* within the ETDRS^71^ fields and has been previously validated in this cohort^70^. We examined three compartments in each B-scan: (1) Inner retinal thickness (IRT), from the internal limiting membrane (ILM) to the interface between the inner nuclear layer and outer plexiform layer (INL/OPL); (2) Outer retinal thickness (ORT), from INL/OPL to Bruch’s membrane (BM) and (3) Total Retinal Thickness (TRT), from ILM to BM. On the en-face ETDRS grid, we used only the central and inner ring subfields, excluding the outer ring due to its higher sensitivity to poor fixation, which often resulted in missing participant data. We analysed each of the five subfields individually (inner-superior, inner-nasal, inner-inferior, inner-temporal, and central subfield) and their average for the ORT measurements. The same approach was applied to IRT and TRT, except the central subfield was excluded for these layers because it is typically absent in the fovea. We investigated the mentioned compartments within the five subfields of the inner 3 mm ETDRS ring: central, inner superior, inner nasal, inner inferior and inner temporal.

**Fig. 5:**
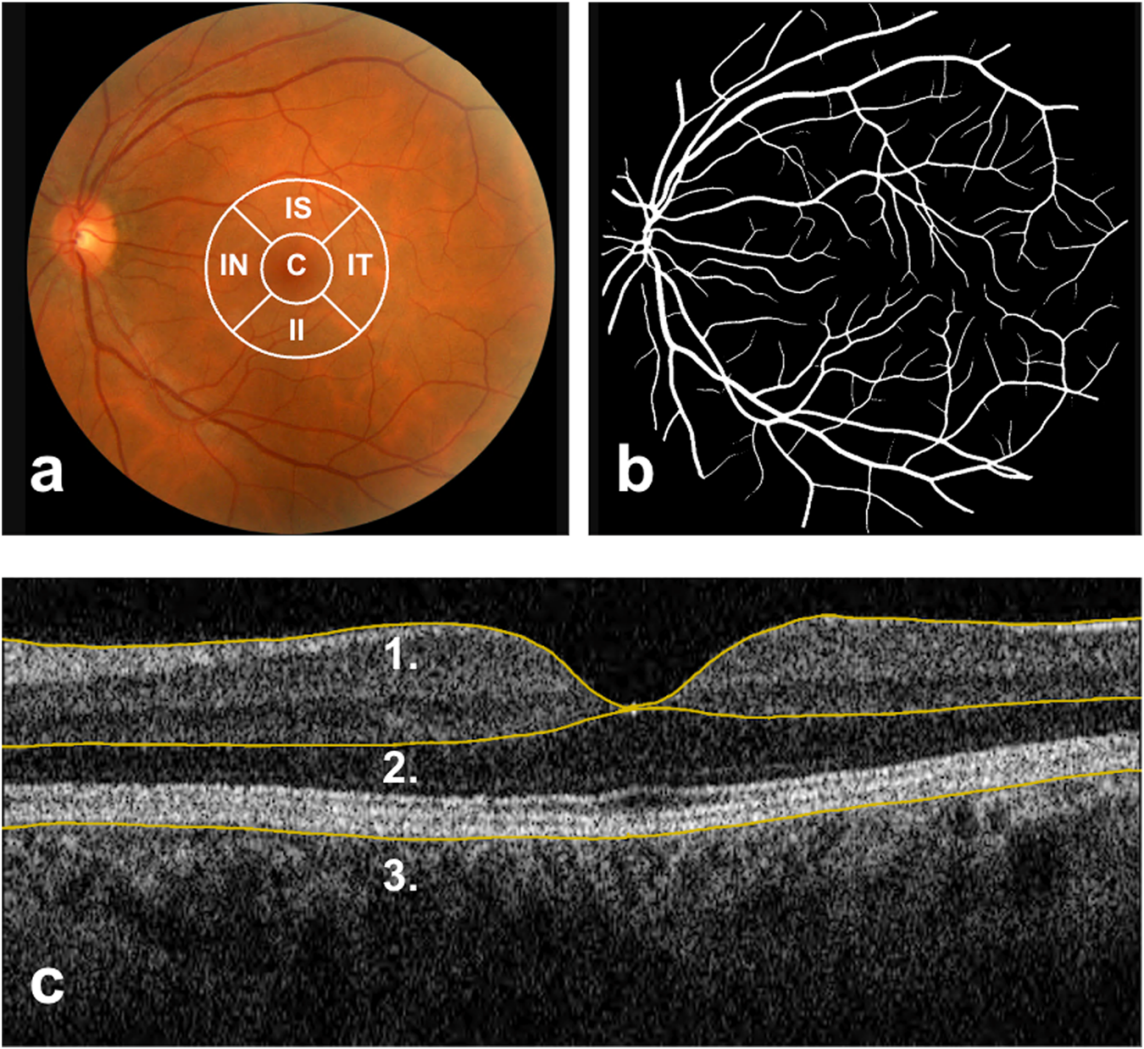
Retinal imaging and segmentation in UKBB. **a** Colour Fundus Photograph (CFP) of the posterior pole, centred on the macula and including the optic disc and temporal vascular arcades. Represented in white at the centre are the inner circles of the Early Treatment Diabetic Retinopathy Study (ETDRS) zones, overlaid on a CFP for demonstration purposes. Typically, the ETDRS grid is superimposed on a macula-centred infrared image. C central subfoveal field, IS inner superior field, IT inner temporal field, II inner inferior fields, IN inner nasal field. **b** Black and white segmentation mask of retinal vessels generated by AutoMorph. **c** Horizontal B-scan OCT over the fovea. Number 1 points to the top segmentation line in yellow, representing the inner limiting membrane; 2 points to the middle segmentation line on the interface between the outer plexiform and inner nuclear layer, and 3 points to the outer edge of the Retinal Pigment Epithelium (RPE). Inner retinal thickness is measured from line 1 to line 2, outer retinal thickness from line 2 to line 3, and total retinal thickness from line 1 to line 3. OCT segmentation measures were automatically calculated by TABS software.

Retinal vascular morphometric characteristics were obtained from CFP using AutoMorph^43^, a deep learning-based tool. The extracted macrovascular parameters, representing the larger retinal vessels, included the central CRAE and CRVE in μm, which reflect standardised vessel calibres for the central retinal artery and vein, calculated using the Knudtson formula. To inform on microvasculature, which pertains to the network of smaller blood vessels like capillaries, arterioles and venules, AutoMorph calculated arteriolar and venular perfusion density and fractal dimension (Minkowski–Bouligand dimension). Perfusion density indicates the ratio of vessel area to the entire image, and fractal dimension reflects vessel complexity and branching. Figure 5 portrays an example of a CFP, inner ETDRS grid, vessel segmentation by AutoMorph and OCT B-scan boundaries from a UKBB participant.

### Covariates and confounding variables

A previous study on macular thickness in the UKBB identified significant associations between central macular thickness and several factors, including older age, female sex, greater myopia, smoking, BMI, and white ethnicity (all *P* < 0.001)18. Based on these findings, we included these factors as covariates in our analysis, along with deprivation. Instead of adjusting for high myopia, we added spherical equivalent <-6D as an exclusion criteria since it affects retinal image magnification. Additionally, we did not adjust for sex because our study exclusively included female participants. Age was calculated from the self-reported date of birth and assessment date and treated as a continuous variable. Ethnicity was self-reported by participants, and their ethnic backgrounds were aggregated into four categories: Black, Other, South Asian, and White. Sex was also self-reported as female or male, while gender was not collected in the UKBB questionnaires. Socioeconomic status was calculated based on the Townsend Deprivation Index and treated into deciles^72^. Smoking status was assessed through pack years of smoking, and BMI was calculated based on measurements obtained at the assessment centre by dividing the mass in kilogrammes by the square of height in metres, both variables continuous. Blood pressure was assessed following 5 min of seated rest, recorded at two consecutive times with a 1-minute interval, utilising the Omron 705 IT electronic blood pressure monitor (OMRON Healthcare). We used the mean of both automated readings, in a continuous fashion. Information regarding systemic diseases was obtained from self-report questionnaires and/or ICD-10 codes, and recorded as present when the questionnaire answer or ICD-10 code was available and positive. A detailed list of all the UKBB showcase fields utilised in this analysis can be found in the Supplementary Table 1.

### Statistical analysis

Questionnaire responses indicating “don’t know” or “prefer not to answer” were treated as missing data. Continuous variables were reported as mean ± SD and range for the baseline unadjusted analysis comparing follicular and luteal phase participants. This analysis was made on a participant level, and eye variables were averaged for each woman. Categorical variables were reported as the number of observations and their respective percentage. We employed the two-sided Wilcoxon-Mann-Whitney test for group comparisons of continuous variables and the U statistic permutation test of independence for group comparisons of categorical variables.

Multivariable linear mixed models were applied to examine the association of the follicular and luteal phases with various retinal parameters extracted from OCT and CFP. We included both eyes from a participant when available, and a random intercept at the participant level allowed for individual variability, keeping the analysis at a participant level. The models were fitted using maximum likelihood estimation and adjusted for age, ethnicity, deprivation, smoking, BMI. Degrees of freedom were estimated using Satterthwaite’s approximation. We report mean ± SD and 95% confidence intervals (CIs).

Statistical analyses were conducted using R version 4.3.0 (released on April 21, 2023), with the packages tidyverse, lme4, lmerTest, data.table and USP. Statistical significance was set at *P* < 0.05.

## Supplementary information

Supplementary information

## Data Availability

The data used in this study originate from the UK Biobank study, which is a prospective study in the United Kingdom that made the data available for researchers around the world, following application. The UKBB is a prospective, population-based, multi-centric cohort data resource with approximately 500,000 participants aged 40–69 years at recruitment, residing in the United Kingdom and registered with the National Health Service (NHS). A subset of these participants underwent ophthalmic imaging and assessment, and their data were included in our study. Detailed information about the study cohort is available on the UKBB website (www.ukbiobank.ac.uk). The North West Multi-center Research Ethics Committee approved the study (reference no., 06/MRE08/65), in accordance with the principles of the Declaration of Helsinki.
