# Supplementary material for: Retinal morphology across the menstrual cycle: insights from the UK Biobank
Source: NPJ Womens Health. 2024 Nov 8;2(1):38. doi: 10.1038/s44294-024-00042-y (PMC11627222; doi:10.1038/s44294-024-00042-y)
Supplement: Supplementary file 1 — Supplementary information [file 44294_2024_42_MOESM1_ESM.pdf]

Supplementary Table 1

UKBB Showcase Fields

Variables and corresponding UKBB data fields used for inclusion/exclusion/analysis. Self-reported were assessed via an interactive questionnaire completed at baseline enrolment into the UK Biobank. Further information can be found at https://biobank.ndph.ox.ac.uk/showcase/

**Variable**	**UK Biobank Data Field**
**Demographic Information**	
Time of assessment	**53:** Date of attending assessment centre<br>**21003:** Age when attended assessment centre
Ethnicity	**21000:** Ethnic background
Index of Multiple Deprivation	**26410:** Index of Multiple Deprivation (England)<br>**26427:** Index of Multiple Deprivation (Scotland)<br>**26426:** Index of Multiple Deprivation (Wales)
Sex	**31:** Sex
**Systemic assessment**	
Diabetes mellitus	**2443:** Diabetes diagnosed by a doctor<br>**2976:** Age diabetes diagnosed
Blood Pressure	**4079:** Diastolic blood pressure, automated reading<br>**4080:** Systolic blood pressure, automated reading
Parkinson's disease (ICD10)	**41270:** Diagnoses - ICD10 (G20)<br>**41280:** Date of first in-patient diagnosis - ICD10 (G20)
Schizophrenia (ICD10)	**41270:** Diagnoses - ICD10 (F20)<br>**41280:** Date of first in-patient diagnosis - ICD10 (F20)
Dementia	**42018**: Date of all-cause dementia report
Smoking	**20161:** Pack years of smoking
Body mass index	**21001:** Body mass index (BMI)
**Ocular assessment**	
Presence of eye disease glaucoma, retinal, injury or macular disease	**6148:** Eye problems/disorders
Best-corrected visual acuity	**5201:** logMAR, final (right)<br>**5208:** logMAR, final (left)
Intra-ocular pressure	**5254:** Intra-ocular pressure, corneal-compensated (right)<br>**5262:** Intra-ocular pressure, corneal-compensated (left)

Spherical Equivalent (SE)<br><br>Formula (per eye):<br>SE = SP + CP/2	**5084**: Spherical power (SP) (right)<br>**5085**: Spherical power (SP) (left)<br>**5086:** Cylindrical power (CP) (left)<br>**5087:** Cylindrical power (CP)(right)
**Female-specific factors**	
Menarche	**2714:** Age when period started (menarche)
Menstrual cycle	**3700:** Time since last menstrual period (days)<br>**3710:** Length of menstrual cycle (days)<br>**3710 = "-6": I**rregular cycle<br>**3720:** Menstruating today
Parity	**2764:** Age at last live birth
Pregnancy	**3140:** Pregnant
Oral contraception	**6153 = "5":** Oral contraceptive pill or minipill<br>**2804 = "-11":** Still taking the pill
Hormonal Replacement Therapy	**6153 = "4":** Hormone replacement therapy
Oestradiol (pmol/L)	**30800**: Oestradiol

Supplementary Table 2

Association between OCT-derived total retinal, inner retinal, and outer retinal thicknesses with cycle phase within the inner 3mm ETDRS ring subfields

Subfields of the Inner 3 mm ETDRS ring	TRT		IRT		ORT	
	Thickness difference, µm, (95% CI)	p-value	Thickness difference, µm, (95% CI)	p-value	Thickness difference, µm, (95% CI)	p-value
**Central**	-	-	-	-	0.12 (-2.99, 3.23)	0.94
**Inner superior field**	3.14 (-0.99, 7.23)	0.14	2.76 (-1.10, 6.62)	0.17	1.21 (-1.53, 3.95)	0.39
**Inner nasal field**	1.40 (-2.48, 5.27)	0.48	1.02 (- 2.82, 4.85)	0.61	1.84 (-1.66, 5.34)	0.31
**Inner inferior field**	0.87 (-2.76, 4.49)	0.64	0.67 (-3.59, 4.93)	0.76	1.10 (-2.81, 5.01)	0.58
**Inner temporal field**	2.24 (-2.76, 4.49)	0.2	1.71 (-1.64, 5.05)	0.32	0.94 (-2.18, 4.06)	0.56

The analysis was adjusted for covariates and estimated through multivariable linear mixed-effects models. Coefficients are presented for the luteal phase, with the follicular phase set as a reference.
Abbreviations: ETDRS = Early treatment diabetic retinopathy study; CI = confidence interval, TRT = total retinal thickness, IRT = inner retina thickness, ORT = outer retina thickness.
